# Guy's Hospital

**Published:** 1829-12

**Authors:** 


### GUY'S HOSPITAL.

### *Malformation of the Genitals—Hermaphrodism.**

An individual exhibiting this unfortunate irregularity of structure was admitted into the Charity ward, September 39th, under the

* The term "hermaphrodism" may be justified in its application to such cases by common parlance, but it is misapplied. An hermaphrodite implies a non-natural creature, capable of performing the generative functions of both sexes. In these instances the individual can perform neither the sexual duties of man or woman. No well authenticated case of human hermaphrodism, in the proper acceptation of the term, has ever been recorded. It is true there is no danger, in the present day, of our following the custom of the Athenians and Romans, of destroying reputed hermaphrodites; but it is incumbent upon us, as men of science, to correct, not to adopt, the popular abuse of words.—EDITORS.

care of Dr. Bright. She was then suffering under a severe form of fever, which rendered her constantly delirious, and in a few days proved fatal.

On her admission,* and more especially when, in order to apply a blister to her head, it was exposed and shaven, every one was struck with the coarse and masculine expression of her countenance: this, and her somewhat square and muscular figure, were all the observations relating to her sex that were made during life; but the post-mortem inspection disclosed the following appearances:

A body analogous to the penis was observed immediately beneath the pubic arch, not free or pendant, but bound down towards the perineum: its length was about two and a half inches, and it terminated in a somewhat bulbous extremity, a little like the glans, but without the usual delicacy of cutaneous organization, without any perforation for the urethra, and without a prepuce. On each side of this body there was a considerable fulness of the integuments, at first view resembling the female labia, but in reality analogous to the male scrotum, as, like it, they each contained a small testis. This separation, into its two halves, of the scrotum, depended on the penis being bound down in the median line, as previously described. The testes were in size like those of a boy six or eight years old, and were connected with vasa deferentia, which were found pervious, and considerably enlarged towards their termination. The vesiculæ seminales were very small; the prostate gland also was remarkably small, and was covered on its sides by a pair of peculiar muscles passing from the rectum to the neck of the bladder. The urethra terminated in the perineum, about one inch from the end of the supposed penis; and half an inch farther there was a blind opening, which fancy might call the rudiment of a vagina, but which was probably nothing more than an enlarged lacuna. The tunica vaginalis was continued some distance up the cord, but at the ring was quite closed. There was a very minute trace of cremaster muscle. The pelvic viscera had no female character whatever, and the formation of the pelvis itself approached to the male rather than to the female standard. The mammæ were considerably developed, but would have been thought small for a healthy female. The lips and chin were clothed with a few scattered, irregular, curling hairs, not more than are often seen on aged females. The outline of the figure, in its muscular developement, squareness, and largeness of limbs, &c. was decidedly more male than female. The cerebellum was natural in structure, and, if it differed at all from the usual developement, was rather small, but this was by no means distinct. No other peculiarities, either diseased or congenital, were observed in any part of the body.

* Speaking of her as a patient, we adhere to the sex then assumed. She was admitted as Mary Cannon, æt. fifty-five or sixty.—*Med. Gaz.*

It appears that, in the former part of her life, this hybrid had assumed the dress and habits of a man; at one time working in a brickyard, at another acting as a groom; then as a milkman; and afterwards she kept a green-grocer's shop. Her habits and manners were rude and bold, sometimes indicating a degree of derangement; more than once she engaged with success in pugilistic encounters; and it is said manifested still less equivocally male propensities. For the last seven or eight years she has appeared as a female, calling herself Mary Cannon; and it is odd enough that she first sustained her new sex at a public-house, called "The World turned upside down," where she engaged herself as "maid of all work." She was not, however, fully received by her female fellow-servants as one of them; suspicion hung about her, and care was always taken to provide for her a separate bed.—*Ibid.*

---

